# Readmissions after elective orthopedic surgery in a comprehensive co-management care system—a retrospective analysis

**DOI:** 10.1186/s13741-021-00218-z

**Published:** 2021-12-15

**Authors:** Felix Rohrer, David Haddenbruch, Hubert Noetzli, Brigitta Gahl, Andreas Limacher, Tanja Hermann, Jan Bruegger

**Affiliations:** 1 Department of Internal Medicine, Sonnenhofspital, 3006 Bern, Switzerland; 2 Centre Hospitalier Universitaire Vaudois, CHUV, 1011 Lausanne, Switzerland; 3 University of Bern, 3012 Bern, Switzerland; 4 Orthopaedie Sonnenhof, 3006 Bern, Switzerland; 5 Clinical Trials Unit (CTU) Bern, University of Bern, 3012 Bern, Switzerland; 6 Stiftung Lindenhof, Campus SLB, Swiss Institute for Translational and Entrepreneurial Medicine, 3010 Bern, Switzerland; 7 University of Zurich, 8006 Zurich, Switzerland

**Keywords:** Unplanned return to operating room, Readmission, Orthopedic surgery, Co-management model

## Abstract

**Background:**

No surgical intervention is without risk. Readmissions and reoperations after elective orthopedic surgery are common and are also stressful for the patient. It has been shown that a comprehensive ortho-medical co-management model decreases readmission rates in older patients suffering from hip fracture; but it is still unclear if this also applies to elective orthopedic surgery. The aim of the current study was to determine the proportion of unplanned readmissions or returns to operating room (for any reason) across a broad elective orthopedic population within 90 days after elective surgery. All cases took place in a tertiary care center using co-management care and were also assessed for risk factors leading to readmission or unplanned return to operating room (UROR).

**Methods:**

In this observational study, 1295 patients undergoing elective orthopedic surgery between 2015 and 2017 at a tertiary care center in Switzerland were investigated. The proportion of reoperations and readmissions within 90 days was measured, and possible risk factors for reoperation or readmission were identified using logistic regression.

**Results:**

In our cohort, 3.2% (42 of 1295 patients) had an UROR or readmission. Sixteen patients were readmitted without requiring further surgery—nine of which due to medical and seven to surgical reasons. Patient-related factors associated with UROR and readmission were older age (67 vs. 60 years; *p* = 0.014), and American Society of Anesthesiologists physical status (ASA PS) score ≥ 3 (43% vs. 18%; *p* < 0.001). Surgery-related factors were: implantation of foreign material (62% vs. 33%; *p* < 0.001), duration of operation (76 min. vs. 60 min; *p* < 0.001), and spine surgery (57% vs. 17%; *p* < 0.001). Notably, only spine surgery was also found to be independent risk factor.

**Conclusion:**

Rates of UROR during initial hospitalization and readmission were lower in the current study than described in the literature. However, several comorbidities and surgery-related risk factors were found to be associated with these events. Although no surgery is without risk, known threats should be reduced and every effort undertaken to minimize complications in high-risk populations. Further prospective controlled research is needed to investigate the potential benefits of a co-management model in elective orthopedic surgery.

## Introduction

No surgery is without risk. Regardless, readmissions and unplanned return to operating room (UROR) are physically and psychologically stressful for the patients and should be prevented as much as possible. In this respect, orthopedic surgery is significant as injuries and disease of the musculoskeletal system are among the most frequent reasons for hospitalization and surgery in Switzerland (Bf, 2019a). Indeed, 30-day readmission rates are reported between 4 and 7% (Schairer et al., 2014a; Schairer et al., 2014b; Bernatz et al., 2015; Lee et al., 2018; Bernatz & Anderson, 2015), while 90-day readmissions lie somewhere between 3 and 25% (Schairer et al., 2014a; Schairer et al., 2014b; Ilyas et al., 2019; Akamnonu et al., 2015; Baaj et al., 2017). Last but not least, the new diagnostic-related groups (DRG) consider readmission within the first 2 weeks following hospital discharge to be part of the first index case—with no new reimbursement due. As a result, unplanned readmissions and reoperations within this time frame also result in financial losses. Jencks et al. estimated that unplanned rehospitalizations in the USA in 2004 generated Medicare costs of up to $17.4 billion (Jencks et al., 2009). However, not every readmission should be classified as a complication. Some can always be expected, and complications or readmissions are not implicitly due to surgeon error (Sokol & Wilson, 2008; Adar et al., 1982).

Several factors are reported to be associated with readmission including length of hospital stay, electrolyte disorders, cardiac valve diseases, diabetes mellitus with end-organ-complication, depression, bleeding disorders, higher American Society of Anesthesiologists physical status (ASA PS) score, and higher body mass index (BMI) (Schairer et al., 2014a; Schairer et al., 2014b; Bernatz et al., 2015; Lee et al., 2018; Bernatz & Anderson, 2015). In cases of elective surgery, one can time the operation to coincide with the patient’s best possible state of health. It is therefore crucial to identify factors which can be optimized to reduce readmissions and minimize complications.

Most readmission studies concentrate on one specific orthopedic field (e.g., spine, knee, hip) (Schairer et al., 2014a; Schairer et al., 2014b; Lee et al., 2018; Bernatz & Anderson, 2015; Ilyas et al., 2019; Akamnonu et al., 2015), or on one single orthopedic procedure (e.g., total knee arthroplasty, total hip arthroplasty) (Schairer et al., 2014a; Schairer et al., 2014b; Ali et al., 2017). They also differ in the period of time examined as many focus on readmissions within 30 days (Bernatz et al., 2015; Lee et al., 2018; Bernatz & Anderson, 2015; Ali et al., 2017).

At our institution, a comprehensive ortho-medical co-management model was implemented in which orthopedic patients with certain comorbidities (cardiac disease, diabetes, kidney insufficiency, chronic pulmonary disease, or dementia) were followed daily by internal medicine specialists during the hospitalization. Furthermore, there was an evaluation on the day of admission by an internal physician, in general 1 day prior to surgery. Patients before hospitalization are generally managed by the general practitioner. Such a model can result in lower rates of postoperative complications after elective hip and knee arthroplasty (Huddleston et al., 2004), and also shortened the average length of hospital stay in older patients with hip fracture (Baroni et al., 2019; Friedman et al., 2009; Bracey et al., 2016; Della Rocca et al., 2013; Phy et al., 2005). Some studies on readmission rates in co-management models for older patients with hip fractures report: decreased rehospitalization to medical wards within 6 months (Fisher et al., 2006) and lower rates of readmission within 30 days (Stephens et al., 2019). Others, however, found no difference in 30-day readmissions (Friedman et al., 2009; Bracey et al., 2016; Phy et al., 2005). To our knowledge, no study has evaluated readmission rates after broad elective orthopedic surgery in a co-management care model to date.

We therefore designed a study, firstly to assess the rates and reasons for UROR during initial hospitalization or readmission within 90 days in a broad elective orthopedic population; and secondly, to identify risk factors associated with UROR and readmission.

## Methods

### Study design

This study is a retrospective analysis of 1295 patients who underwent general elective orthopedic surgery between November 2015 and November 2017 at one tertiary care center in Bern, Switzerland. This is a sub-study of the prospective randomized control trial DECO-SSI (DECOlonization and SSI) (Rohrer et al., 2020). Of the initial study population (*N* = 1318), 23 patients were excluded: 14 were lost to follow-up at 90 days, 7 were excluded for canceled surgery, and 2 withdrew their consent. The study protocol was approved by the local Ethics Committee (PB_2016_00256).

### Data collection

Patient characteristics and the occurrence of unplanned reoperations and readmissions were surveyed prospectively. Other data (readmission date, number of readmissions, reason for readmission) were retrospectively extracted from the KISIM (Cistec AG, Zurich, Switzerland) electronic patient file system. All relevant data was entered into the secure web data storing system Research Electronic Data Capture (REDCap, Version 8.5.19, Vanderbilt University, Nashville, Tennessee, USA).

### Participants

This study explored the population of an earlier randomized controlled trial (RCT) (Rohrer et al., 2020). All patients scheduled for elective orthopedic surgery were evaluated for eligibility. Patients older than 16 years were included if they had signed written informed consent at least 14 days before operation. Patients with allergy to mupirocin or chlorhexidine, any foreign nasal body, pregnancy, or on-going intervention for a documented infection were excluded.

Any readmissions and URORs were captured at 30 days and 90 days following surgery via phone interview and review of electronic patient charts. Readmissions to the same hospital (or any other hospitals) were recorded and subdivided into those with or without surgery. For each reoperation, we investigated if it was in the same surgical field and whether there was a possible relation to the index operation. Similarly, readmissions without surgery were analyzed for the reason (surgical or medical) and whether there was a certain, possible, or unlikely relation to the initial operation. Reoperations on the same surgical site were always classified as “certain.” Causes of UROR were divided into subgroups as follows: surgical site infection, trauma, hemorrhagic complications (hematoma, bleeding), delayed healing, stiffness (limited mobility needing intervention), mechanical complications (junctional/secondary fracture, junctional kyphosis, etc.), or technical problems during primary surgery (material requiring rapid removal, etc.) liable to have contributed to UROR (Pujol et al., 2015). The allocation was performed by two internal medicine physicians and one orthopedic surgeon.

### Outcomes

Firstly, the number of UROR during initial hospitalization and readmissions within 90 days was measured. If the same patient had been readmitted several times, they were regarded as one single case. Secondarily, reasons for readmission were collected and their relation to initial surgery determined. Finally, the following predefined factors were compared between patients without event and those with an event. Patient characteristics included age, sex, active smoker, regular alcohol consumption, BMI, chronic obstructive pulmonary disease (COPD), liver disease, congestive or ischemic heart disease, renal insufficiency, diabetes mellitus, transient ischemic attack (TIA) or cerebrovascular insufficiency (CVI), and ASA PS score. Surgical characteristics included type of main procedure (spine; pelvic, hip, or upper extremity; knee or foot), use of foreign material (metal or inert synthetics) or prosthetic surgery, and duration of operation.

### Statistical methods

Statistical analysis was performed by a statistician from the clinical trial unit (CTU), University of Bern, Switzerland. We used logistic regression including patient age, ASA PS score, duration of surgery, and surgical procedure site as covariates to investigate the risk of unplanned return to operating room or hospital. Model fit was checked using the Hosmer-Lemeshow goodness-of-fit test. We explored implantation of foreign material as covariate in the logistic model but decided against it because it did not improve the model fit. Continuous variables were shown as median with quartiles after checking distribution and comparisons were made using Mann-Whitney test. Categorical data are shown as percent (%) and were compared using Fisher’s exact test. All analyses were carried out using Stata 16 (Stata Corp., College Station, Texas). A *p* value < 0.05 was considered to be statistically significant.

## Results

**Fig. 1:**
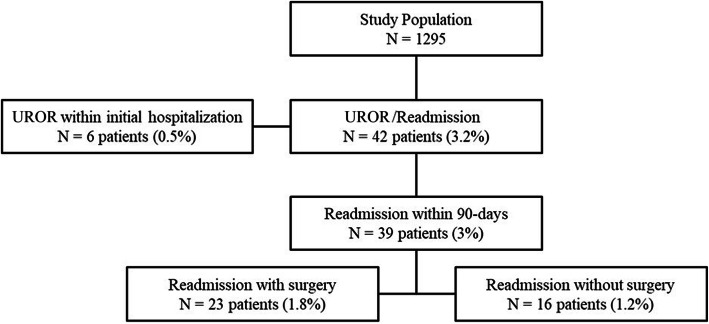
Flowchart of the study

UROR during initial hospitalization or unplanned readmission with or without UROR within 90 days of discharge occurred 48 times in 3.2% of patients (42 of 1295) (Figure 1).

UROR within initial hospitalization occurred in 0.5% (6 of 1295 patients), five of which were in the same surgical field and one in another surgical field (hemicolectomy due to Ogilvie’s syndrome after spine stabilization surgery). Causes for UROR in same surgical field within initial hospitalization were hemorrhagic (4 of 5 UROR) and technical (1 of 5 UROR). Later readmission occurred in three patients with UROR during initial hospitalization. UROR at same operation site within 90 days affected 1.7% of patients (22 of 1295). Two were necessary in one patient. The causes were 60.1% mechanical (14 of 23 UROR), 8.7% hemorrhagic (2 of 23 UROR), and 4.4% SSI, stiffness, or technical problems (1 of 23 each). Trauma accounted for 17.4% (4 of 23 UROR). Lumbar decompression surgery for a herniated disc which occurred 9 weeks after knee arthroplasty was required in 1 patient; assessment of causality was possible due to the temporal relationship. UROR and readmissions are shown in Table 1. Readmission without needing surgery occurred in 1.2% patients (16 of 1.295): 56.3% (9 of 16) for medical reasons and 43.7% (7 of 16) due to surgical reasons. The relation to the index surgery was “certain” in 11 cases, and “possible” in five cases. The reasons are shown in Table 2. Comparison of patients with and without UROR is shown in Table 3. Patients with unplanned readmission compared to patients without readmissions were older (67 vs. 60 years; *p* = 0.014), had a higher ASA PS score ≥ 3 (43% (18 of 42 patients) vs. 18% (223 of 1253 patients); *p* < 0.001), underwent more often spine surgery (57% (24 of 42 patients) vs. 17% (215 of 1253 patients); *p* < 0.001), had more foreign material implanted (metal or non-absorbable synthetic material) (62% (26 of 42 patients) vs. 33% (410 of 1253 patients); *p* < 0.001), and duration of operation was longer (76 min vs. 60 min; *p* <0.001).

**Table 1:** Unplanned readmission and UROR during initial hospitalization

Total	*N* = 42 patients
UROR during initial hospitalization	6 (14%)
UROR during later hospitalization at same operation site	22 (52%)
UROR during later hospitalization at other operation site, but possibly related to initial operation	1 (2.4%)
Readmission without surgery, but related to initial operation	
Certain	11 (26%)
Possible	5 (12%)

*Note*: two patients who underwent UROR during initial hospitalization also underwent UROR at the same site during a further admission, and one further patient was re-admitted without surgery but with certain causal relation to initial operation

**Table 2:** Reasons for readmissions without surgery

Medical (*N* = 9)		
	Certain correlation	Pulmonary embolism (*N* = 2) Others (*N* = 2) (Constipation; hypertensive crisis)
	Possible correlation	Drug problems (*N* = 2) Others (*N* = 3) (Cardiac decompensation; Vertigo; Stroke)
Surgical (*N* = 7)	Certain correlation	Pain (*N* = 5) Hematoma/Seroma (*N* = 2)

**Table 3:** Patient and surgical characteristics

	Total, *N* = 1295	Readmissions/UROR *N* = 42	No return, *N* = 1253	*p*
Age (years)	61 [50, 69]	67 [54, 72]	60 [50, 69]	0.014
Sex (female)	683 (53%)	25 (60%)	658 (53%)	0.43
Active smokers	223 (17%)	12 (29%)	211 (17%)	0.06
Regular alcohol consumption	402 (31%)	11 (26%)	391 (31%)	0.61
BMI (kg/m^2^)	26 [24, 30]	26 [24, 30]	26 [24, 30]	0.84
COPD	23 (1.8%)	4 (10%)	19 (1.5%)	0.005
Liver disease	10 (0.77%)	1 (2.4%)	9 (0.72%)	0.28
Congestive or ischemic heart disease	83 (6.4%)	4 (10%)	79 (6.3%)	0.34
Renal insufficiency	11 (0.85%)	0 (0.00%)	11(0.88%)	1.00
Diabetes	81 (6.3%)	3 (7.1%)	78 (6.2%)	0.74
TIA/CVI	45 (3.5%)	4 (10%)	41 (3.3%)	0.05
ASA PS score				0.001
1	443 (34%)	12 (29%)	431 (34%)	
2	609 (47%)	12 (29%)	597 (48%)	
3	241 (19%)	18 (43%)	223 (18%)	
4	1 (0.08%)	0 (0.00%)	1 (0.08%)	
Type of procedure				< 0.001
Spine	239 (18%)	24 (57%)	215 (17%)	
Pelvic/Hip or upper extremity	524 (40%)	6 (14%)	518 (41%)	
Knee or foot	532 (41%)	12 (29%)	520 (42%)	
Foreign material				< 0.001
Prosthetic surgery	613 (47%)	12 (29%)	601 (48%)	
Metal or non-absorbable synthetic material	436 (34%)	26 (62%)	410 (33%)	
No foreign material used, no foreign material left in surgical site or absorbable synthetic material used	243 (19%)	3 (7.1%)	240 (19%)	
Duration of operation (in minutes)	60 [43, 87]	76 [60, 130]	60 [43, 86]	< 0.001
Duration of initial hospitalization (days)	5.0 [4.0, 8.0]	6.5 [5.0, 9.0]	4.0 [2.0, 5.0]	< 0.001

**Fig. 2:**
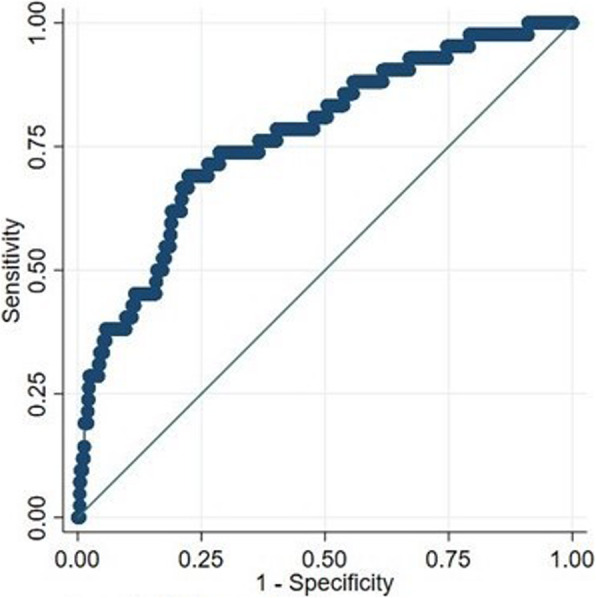
ROC curve. Area under the ROC curve was 77%, 95% confidence interval 69 to 85%

In multivariable analysis, spine surgery was highly associated with the risk of unplanned readmission, even when adjusted for patient age, ASA PS score, and duration of operation (Table 4). The adjusted odds ratio (OR) of unplanned return after spine surgery was 6.6 (95% CI: 2.5–17.7, *p* < 0.001); compared to that for pelvis, hip, or upper extremities. The model consisting of four variables (age, ASA PS score, duration of operation, spine surgery) showed moderate to high discriminative power (AUROC 0.8, 95% CI 0.7 to 0.9, see Fig. 2) to predict how likely a patient is to return to operating room or hospital. There was no case of a so-called never event of surgery (retained foreign body, wrong site, wrong patient, or wrong procedure).

**Table 4:** Outcome analysis (multivariable model)

	OR (95% CI)	*p*
Age per 10 years	1.08 (0.82 to 1.42)	0.605
ASA PS score	1.53 (0.99 to 2.38)	0.057
Duration of operation (per 10 min)	1.06 (1.00 to 1.13)	0.063
Pelvic/Hip or upper extremity	Reference	
Spine surgery	6.52 (2.43 to 17.51)	< 0.001
Knee or foot surgery	1.86 (0.68 to 5.09)	0.225

*Note* that surgery on pelvis, hip, or upper extremity was selected as reference category of surgical sites, leading to less frequently unplanned returns

## Discussion

### Readmission rates

UROR and readmissions affect patients negatively and are associated with worse overall outcomes. This is especially of importance in orthopedic procedures as they are the most common surgeries performed (AHRQ AfHRaQ, 2014). Co-management care systems have been shown to decrease complications and readmission rates in patients with hip fracture; but no data exists regarding readmission rates in elective orthopedic surgery. Comparing our results to literature is therefore difficult, but our findings (3.2% readmission and UROR proportion) seem to be lower than reported elsewhere. Examples from the literature report 90-day readmission rates ranging from 3.3 to 24.8 % after spine surgery (Ilyas et al., 2019; Akamnonu et al., 2015; Baaj et al., 2017), 8.4% in total knee arthroplasty (Schairer et al., 2014b), and 8.8% after total hip arthroplasty (Schairer et al., 2014a).

Our study found that readmissions due to medical reasons accounted for 21.4% of all readmissions compared to the 26.4% of Bernatz et al. (Bernatz & Anderson, 2015), and the 26% reported by Dailey et al. (Dailey et al., 2013). Interestingly, while 19.4% of readmissions found by Dailey et al. were due to infections other than surgical site infection (SSI), we registered zero cases due to other infections (Dailey et al., 2013). Our rate of pulmonary embolism, on the other hand, is similar to that described in the literature (4.8% vs. 3.6% from Dailey et al. (Dailey et al., 2013)).

Other cofounders such as patient characteristics could possibly have influenced readmission and UROR rates. For example, patients in the Ilyas et al. study were older (67 vs. 60 years), smoked more (61.6% vs. 17%), had higher rates of coronary artery disease (CAD)/chronic heart failure (CHF) (23.6%, 7.8 % respectively vs. 6.4%), and diabetes (26.9% vs. 6.3%) than in our study (Ilyas et al., 2019). Similarly, Lee, Dailey, and Ali reported more diabetics (Lee et al., 2018; Ali et al., 2017; Dailey et al., 2013); and 61% of patients in the study of Lee et al. also suffered from cardiac comorbidities (Lee et al., 2018). In comparison to Baaj et al.—who found a readmission rate of 24.8%—our study population was slightly older (61 vs. 55.5 years), but had less coronary artery disease, chronic renal failure, diabetes mellitus, and contained fewer smokers (Baaj et al., 2017).

Many studies describe SSIs as of the leading causes for readmission for surgical reasons (Schairer et al., 2014a; Schairer et al., 2014b; Bernatz et al., 2015; Lee et al., 2018; Bernatz & Anderson, 2015; Ilyas et al., 2019; Baaj et al., 2017; Pujol et al., 2015; Dailey et al., 2013), but our center’s SSI rate was unexpectedly low at 0.3%. Data from the Swiss nosocomial infection surveillance program from 2016 to 2017 showed SSI rates of 1.1% in elective prosthetic hip surgery, 0.8% in prosthetic knee surgery, and 1.9% and 1.2% in laminectomy, with and without implantation of foreign material (Marie-Christine Eisenring et al., 2019). Several factors could have contributed to our low SSI rate. Participation in the surveillance program is voluntary (Geubbels et al., 2006; Mabit et al., 2012; Staszewicz et al., 2014; Haley et al., 1985; Abbas et al., 2019; Astagneau et al., 2009) and only elective procedures are included—emergency procedures have higher proportions of SSI (Agodi et al., 2015; Isik et al., 2015). An additional factor contributing to our low proportion of SSIs as well as overall readmission could be that our orthopedic surgeons are highly specialized and only operate in one subspecialty.

In summary, one possible reason for our lower number of medical readmissions could be the co-management system at our institution. However, as this study was not designed to analyze the efficacy of such a system, this remains a hypothesis and warrants further prospective controlled trials.

### Risk factors for UROR and readmissions

We observed that only spine surgery is an independent risk factor for readmission and UROR. Although patient age is typically a confounder for many associations of variables with different kinds of risk, age was not an independent risk factor in our multivariable analysis. Indeed, the ASA PS score as surrogate for comorbidities had a larger OR than age, but was not significant, however close to being significant. In current literature however, ASA PS score greater than 3 is a proven risk factor (Bernatz et al., 2015; Bernatz et al., 2016; Ward & Group RAS, 2019; Keswani et al., 2016; Tayne et al., 2014). Findings concerning the association of age and readmission are inconsistent. Some studies did not report a difference (Schairer et al., 2014b; Lee et al., 2018; Akamnonu et al., 2015; Dailey et al., 2013; Tayne et al., 2014; Chern et al., 2015), while others found a significant association (Ilyas et al., 2019; Baaj et al., 2017; Ali et al., 2017; Keswani et al., 2016; Avram et al., 2014; Hageman et al., 2014; Sherman et al., 2008).

Our data showed that spine surgery is also an independent risk factor for readmission. At 10.5% (24 of 229 patients undergoing spine surgery), the readmission rate was slightly higher than a study by Cui et al. with 26,727 patients which reported a readmission rate of 9.7% within 90 days following posterior lumbar fusion (Cui et al., 2019). On the other hand, Baaj et al. found a readmission rate of 24.8% after lumbar spinal fusion in a 10-year analysis of 86,869 patients (Baaj et al., 2017). However, there are significant differences in the various procedures and studies. Zaki et al. described a readmission rate of 7.7% after anterior cervical discectomy and fusion, and of 16.9% after posterior cervical fusion within 90 days (Zaki et al., 2019). Akamnonu et al. on the other hand, described an overall readmission rate of 3.3% within 90 days. They also found variations between 2.1 and 7.1% depending on pathology (Akamnonu et al., 2015). Hence, the independent association of spine surgery with readmission in our study could be explained due to the lower readmission proportions of the other subspecialties rather than higher readmissions after spine surgery.

We also observed that the duration of operation is significantly associated with readmissions, even if not independently. There is no consensus about this correlation in recent literature. Bernatz et al.’s meta-analysis found that longer spine surgery duration is associated with readmission (Bernatz & Anderson, 2015), which is not entirely surprising as it is a known risk factor for developing SSI (Ilyas et al., 2019).

We also found a link between the implantation of foreign material and higher readmission risk. In the literature, 9–19% readmissions are implant-related (Ilyas et al., 2019; Akamnonu et al., 2015; Pujol et al., 2015). Additionally, SSIs after laminectomy with implantation of foreign material are described to be more common than in laminectomy without implantation (Marie-Christine Eisenring et al., 2019).

Though we found a significant association between readmissions and COPD, the relevance of this result is unclear considering the rather small absolute number of 23 patients (1.8%) with COPD in our study population. This is a smaller proportion compared to a total of 400,000 patients with COPD in Switzerland (4.6% considering a total population of 8,606,033 in 2019 (Bf, 2019b)) according to the Lung League (Lungenliga) (Lungenliga, 2020).

There is no consensus about gender being a risk factor for readmission or not, with various studies finding higher rates in male sex (Keswani et al., 2016; Avram et al., 2014), in female sex (Ilyas et al., 2019; Tayne et al., 2014), or no difference (Dailey et al., 2013; Chern et al., 2015; Avram et al., 2014). Our data shows no association of gender with readmission. Similarly, we did not find a significant correlation of readmission with active smoking (*p* = 0.06). Findings about smoking in literature are inconsistent as well: i.e., Ward et al. were not able to make a conclusive statement about the correlation (Ward & Group RAS, 2019), but Tischler et al. linked active smoking and packs per decade with higher readmission rates (Tischler et al., 2017).

### Strengths and limitations

A key limitation is the observational design of this study, in which correlation between readmission and the comanagement care system remains hypothetical. Nevertheless, we showed that proportions were low, warranting further prospective controlled research to investigate possible correlation. Another relevant point is that this trial’s data originates from one single center.

Analysis was retrospective in nature as “reasons for readmission” were not collected prospectively and had to be retrieved retrospectively from patient charts. Readmissions were prospectively surveyed by phone interview to assure that all readmissions, including to other hospitals, were recorded. Due to this, we may have found such cases that would otherwise have been missed. As mentioned, most studies investigate single orthopedic fields (Schairer et al., 2014a; Schairer et al., 2014b; Lee et al., 2018; Bernatz & Anderson, 2015; Ilyas et al., 2019; Akamnonu et al., 2015) or single orthopedic procedures (Schairer et al., 2014a; Schairer et al., 2014b). Our study is not limited to one orthopedic field or procedure and contributes to a broader collection of readmission data including: prosthetic surgery, operations with or without implantation of foreign material, and various joint and operation sites. Our data is therefore transferable to all elective orthopedic procedures and becomes more clinically valuable.

## Conclusion

Compared to the literature, this study showed low numbers of UROR and readmission up to 90 days post-surgery. Only spine surgery was an independent risk factor for readmissions in our analysis. A co-management system could be considered to optimize perioperative care, but prospective controlled trials to assess the benefit on readmission rates in elective orthopedic surgery are needed. No surgery is without risk, but those known should be reduced as much as possible and every effort undertaken to minimize complications, especially in high-risk patients.

## Data Availability

The datasets used and/or analyzed during the current study are available from the corresponding author upon reasonable request.

## Abbreviations

UROR: Unplanned return to operating room
ASA PS: American Society of Anesthesiologists physical status
DRG: Diagnostic-related groups
BMI: Body mass index
RCT: Randomized controlled trial
COPD: Chronic obstructive pulmonary disease
TIA: Transient ischemic attack
CVI: Cerebrovascular insufficiency
CTU: Clinical trial unit
OR: Odd ratio
SSI: Surgical site infection
CAD: Coronary artery disease
CHF: Chronic heart failure
